# Whole-genome sequencing-based genetic diversity, transmission dynamics, and drug-resistant mutations in *Mycobacterium tuberculosis* isolated from extrapulmonary tuberculosis patients in western Ethiopia

**DOI:** 10.3389/fpubh.2024.1399731

**Published:** 2024-08-09

**Authors:** Basha Chekesa, Harinder Singh, Norberto Gonzalez-Juarbe, Sanjay Vashee, Rosana Wiscovitch-Russo, Christopher L. Dupont, Musse Girma, Oudessa Kerro, Balako Gumi, Gobena Ameni

**Affiliations:** ^1^Aklilu Lemma Institute of Pathobiology, Addis Ababa University, Addis Ababa, Ethiopia; ^2^Collage of Natural and Computational Science, Wallaga University, Nekemte, Ethiopia; ^3^Infectious Diseases, Genomic Medicine, and Synthetic Biology Group, J. Craig Venter Institute, Rockville, MD, United States; ^4^Genomic Medicine, Environment & Sustainability, and Synthetic Biology groups, J. Craig Venter Institute, La Jolla, CA, United States; ^5^Institute of Agriculture, The University of Tennessee, Knoxville, TN, United States; ^6^College of Agriculture and Veterinary Medicine, United Arab Emirates University, Al Ain, United Arab Emirates

**Keywords:** Extrapulmonary tuberculosis, drug resistance-conferring mutations, genetic diversity, *Mycobacterium tuberculosis*, transmission dynamics, whole-genome sequencing

## Abstract

**Background:**

Extrapulmonary tuberculosis (EPTB) refers to a form of Tuberculosis (TB) where the infection occurs outside the lungs. Despite EPTB being a devastating disease of public health concern, it is frequently overlooked as a public health problem. This study aimed to investigate genetic diversity, identify drug-resistance mutations, and trace ongoing transmission chains.

**Methods:**

A cross-sectional study was undertaken on individuals with EPTB in western Ethiopia. In this study, whole-genome sequencing (WGS) was employed to analyze *Mycobacterium tuberculosis* (MTB) samples obtained from EPTB patients. Out of the 96 genomes initially sequenced, 89 met the required quality standards for genetic diversity, and drug-resistant mutations analysis. The data were processed using robust bioinformatics tools.

**Results:**

Our analysis reveals that the majority (87.64%) of the isolates can be attributed to Lineage-4 (L4), with L4.6.3 and L4.2.2.2 emerging as the predominant sub-lineages, constituting 34.62% and 26.92%, respectively. The overall clustering rate and recent transmission index (RTI) were 30 and 17.24%, respectively. Notably, 7.87% of the isolates demonstrated resistance to at least one anti-TB drug, although multi-drug resistance (MDR) was observed in only 1.12% of the isolates.

**Conclusions:**

The genetic diversity of MTBC strains in western Ethiopia was found to have low inter-lineage diversity, with L4 predominating and exhibiting high intra-lineage diversity. The notably high clustering rate in the region implies a pressing need for enhanced TB infection control measures to effectively disrupt the transmission chain. It’s noteworthy that 68.75% of resistance-conferring mutations went undetected by both GeneXpert MTB/RIF and the line probe assay (LPA) in western Ethiopia. The identification of resistance mutations undetected by both GeneXpert and LPA, along with the detection of mixed infections through WGS, emphasizes the value of adopting WGS as a high-resolution approach for TB diagnosis and molecular epidemiological surveillance.

## Introduction

Tuberculosis (TB) stands as the second most prevalent disease worldwide, following closely behind Coronavirus disease (COVID-19). Globally, in 2022, approximately 10.6 million people *fell ill with TB*, reflecting a 4.5% increase from 2020. Among them, 1.30 million individuals *died from TB*, including 187,000 cases among those co-infected with human immunodeficiency virus (HIV) (1). While pulmonary tuberculosis (PTB) primarily affects the lungs, extrapulmonary tuberculosis (EPTB) involves other organs and tissues in the body (2). According to the WHO, EPTB constituted 16% of the new and relapsed TB cases reported worldwide in 2020. In Ethiopia, both forms of TB pose significant public health threats, with the country ranking third worldwide in terms of EPTB cases, surpassing PTB burdens observed in many other regions (3). Notably, in 2020, EPTB accounted for 30% of the reported cases in Ethiopia (4).

TB is instigated by members of the *Mycobacterium tuberculosis* complex (MTBC), which encompasses nine distinct phylogenetic lineages (5): L1 (Indo-Oceanic); L2 (East Asian); L3 (East African-Indian); L4 (Euro-American); L5 (*M. africanum* West-African 1); L6 (*M. africanum* West-African 2); L7 (Ethiopia) (6, 7); L8 (*M. tuberculosis* from the African Great Lakes) (8); and the recently identified *M. africanum* L9 (5). Each lineage has evolved to adapt specifically to diverse human populations, exhibiting global prevalence in some cases and marked geographical restrictions in others (9). Understanding the predominant lineages within a particular region holds significance for TB prevention and care. The strain type also plays a pivotal role in influencing disease outcomes, variations in vaccine efficacy (10), the emergence of drug resistance (11), and the overall epidemiology of the disease (12).

Molecular epidemiology has become increasingly crucial as a tool for efficient TB control, enabling the identification of distinctive strains associated with outbreaks (13), virulence (14), and the development of drug resistance (15). Moreover, molecular epidemiology can elucidate the geographical origin of a strain and unveil new lineages (7). In Ethiopia, the molecular epidemiology of TB has largely depended on spoligotyping. This technique analyzes only a small segment of the MTBC genome, limiting its ability to accurately reconstruct complex transmission chains and establish clear transmission links between patients (16).

Whole-genome sequencing (WGS) is revolutionizing our understanding of drug resistance, clinical management, and transmission patterns, significantly contributing to disease control efforts (17). While a few studies in Ethiopia have recently explored the genetic diversity and drug resistance of MTBC using WGS, none have specifically investigated EPTB patients in the western region of Ethiopia. In western Ethiopia, specifically in western Oromia, a persistent conflict spanning over three decades has significantly undermined TB control programs. This conflict has resulted in challenges such as overcrowding due to social displacement, widespread hunger, and overcrowded prisons. Therefore, examining the genotype of MTB strains becomes crucial for contributing to TB prevention and care initiatives. Consequently, this study was undertaken using WGS to explore the genetic diversity, resistance-conferring mutations, and ongoing transmission of EPTB in western Ethiopia.

## Materials and methods

### Study setting

**Figure 1:**
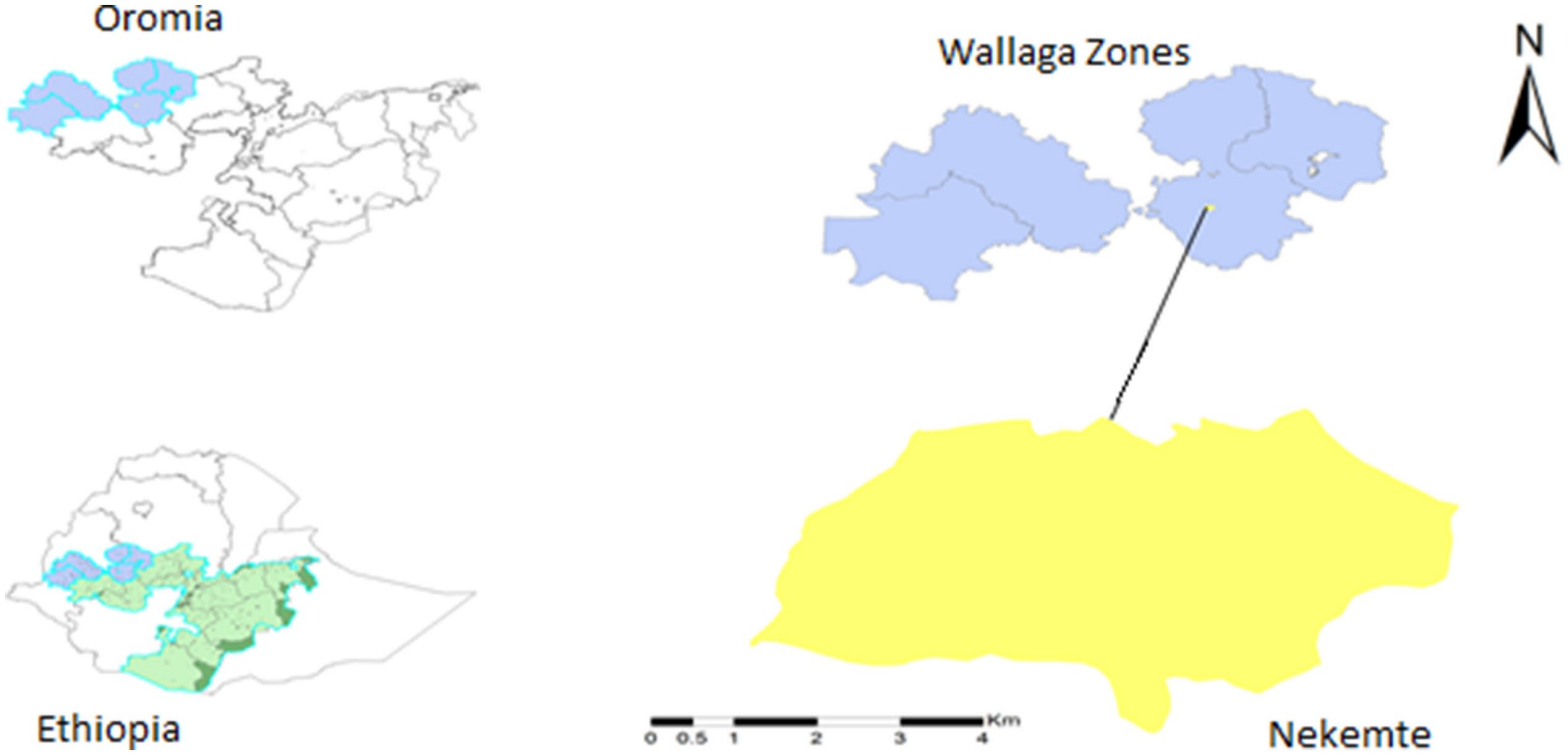
Map of the study area (Nekemte City), western Ethiopia. Patients were recruited from two hospitals within the city: Nekemte Specialized Hospital and Wallaga University Referral Hospital, both located in Nekemte City. These hospitals were selected because they are the only facilities with pathologists serving the four Wallaga Zones and the surrounding areas.

The study took place at Nekemte Specialized Hospital and Wallaga University Referral Hospital, both located in Nekemte City, the capital of East Wallaga Zone (Figure 1), approximately 320 km west of Addis Ababa, Ethiopia. These hospitals were selected because they serve as the sole diagnosis and treatment centers for EPTB in western Oromia, Ethiopia.

An institution-based, cross-sectional study design was conducted on confirmed EPTB patients who visited the listed health facilities between August 2018 and December 2019. Study participants encompassed all age groups, with those unwilling to provide consent or assent being excluded from participation.

### Collecting, transporting, and culturing of the specimens

Following the participants’ consent, 264 fine needle aspiration (FNA) specimens from lymph nodes and other tissues were collected and examined by a pathologist using a microscope. The FNA cytology slides were air-dried, and a Wright stain was applied to the smear for one to 3 min. Subsequently, a second 2 ml of distilled water was added, left to stand for twice the duration of the first phase (2–6 min). After drying, a light microscope was employed for examination. All FNA MTB-positive specimens were meticulously packed in an ice box at +4°C and transported to the Aklilu Lemma Institute of Pathobiology (ALIPB), TB Laboratory, Addis Ababa University (AAU), for screening the growth MTBC.

The culturing of samples adhered to the Petroff procedure and was executed at ALIPB, AAU (18). Specimens were decontaminated by centrifugation at 3,000 rpm for 15 min using a final NaOH concentration of up to 2%, achieved by mixing with an equal amount of 4% NaOH stock solution and the sample. After discarding the supernatant, the sediment was neutralized with 2 N HCl. The inoculation of the sediment occurred on two conventional Löwenstein–Jensen (LJ) egg slant media, with one containing 0.6% sodium pyruvate and the other 0.75% glycerol. The inoculated slant was then incubated at 37°C for a minimum of 4 weeks, with weekly observations of mycobacterial colony growth. A total of 121 culture-positive isolates were harvested and then transported to J. Craig Venter Institute (JCVI), United States after DNA extraction.

### DNA extraction and whole-genome sequencing

DNA extraction was carried out using a modified chloroform and acetyl trimethyl ammonium bromide (CTAB) protocol, as previously detailed (19). Subsequently, the concentration of DNA was quantified using Qubit 4 technology with a Qubit dsDNA HS Assay kit (Thermo Fisher Scientific, Waltham, USA). For the preparation of sequencing libraries, a genomic DNA concentration of 1 ng was utilized, and the Illumina Nextera XT library preparation kit (Illumina, San Diego, USA) was employed following the manufacturer’s instructions. Quality control procedures for the library were implemented using the Agilent High Sensitivity DNA Kit (Agilent, CA, USA) and the Qubit dsDNA HS Assay kit (Thermo Fisher Scientific, Waltham, USA). Following this, libraries were manually normalized based on DNA concentration and the average fragment size of the libraries. Of the 121 culture-positive isolates, WGS was performed on 96 randomly selected isolates at the JCVI laboratory in the United States. This was done using Illumina NovaSeq 6000 technology with 2 × 150 paired-end chemistry, producing paired-end FastQ files (20).

### Bioinformatics analysis

#### Quality check and *de novo* assembly

To ensure data quality, FastQC (v0.12.1) (21) was employed to verify the quality of the raw reads both before and after trimming, which involved removing adapter sequences, low-quality reads, and filtering for a minimum read length. Trimming of the raw FastQ Illumina reads was carried out to eliminate Illumina adapter and low-quality reads using Trimmomatic v0.39 (22) with the following parameters: phred33, LEADING: 3 TRAILING:3 SLIDINGWINDOW:4:15 MINLEN:36. Subsequently, the *de novo* assembly of MTB genomes was executed using SPAdes v3.15.5 (23). Various odd k-mer sizes within the range k = 21 to k = 87 were employed for this assembly process.

### Variant calling

The trimmed paired-end reads of the investigated strains underwent analysis using the MTBseq pipeline (v1.0.4) (24), a semi-automated bioinformatics pipeline specifically designed for the analysis of MTBC isolates. Variant calling, encompassing single nucleotide polymorphisms (SNPs) and insertions/deletions (InDels), was carried out with stringent filtering criteria. These criteria included a minimum coverage requirement of 10 forward reads and 10 reverse reads indicating the allele, a 75% allele frequency, and a minimum of four read calls with a phred score of at least 20. Automatic exclusion criteria were applied for variants that appeared within a 12 bp window in the same isolate, positions in drug resistance-associated genes, those detected in PE/PPE, and sites with ambiguous calls in over 5% of isolates, along with other hard-to-map regions. Datasets with a mean coverage depth below 20x and less than 80% alignment to the reference genome were excluded from further analysis.

### Genomic cluster definition and analysis

Genomic clusters were identified without relying on epidemiological data, defining a cluster as patient isolates exhibiting a genomic difference of 12 or fewer single-nucleotide polymorphisms (SNPs). This threshold of 12 SNPs was established based on prior definitions, serving as the upper limit for genomic relatedness within genome-based clusters (25, 26). In our study, we applied an SNP threshold of 12, and the MTBseq (24) pipeline was employed for cluster analysis (26). The size of a cluster was determined based on the total number of genomes it encompassed, categorized as small (2 genomes), medium (3–5 genomes), or large (>5 genomes). We calculated the clustering rate and RTI using the ‘n method’ (n/N100) and ‘n-1 method’ ((n-c)/N100), respectively. Here, N represents the total number of sampled cases, c is the number of clusters, and n is the total number of cases within the clusters (25).

### *In silico* spoligotyping

For next-generation sequencing reads, *in silico* spoligotyping was conducted using the SpoTyping program version v2.0 (27) with default parameters. The SITVIT2 server was then utilized, based on the identified spoligotypes, to determine the lineage (28). Isolates exhibiting a similar pattern to those in the SITVIT database were assigned a Spoligo International Type (SIT) number. Isolates that did not match any SIT numbers were categorized as “Orphan” spoligotypes.

### *In silico* drug resistance, lineage typing, and mixed infection identification

To identify the MTB species, lineages, sub-lineages, and drug resistance mutations (SNPs, indels, and frameshifts), the isolated strains were analyzed using TB-Profiler v5.0.1 (29). This involved aligning raw paired-end reads against the reference genome MTB H37Rv. The resistance mutations predicted by TB-Profiler were further validated using mykrobe (v0.12.1) (30), along with the results from MTBseq (24), which provides a list of mutations in genes associated with antimicrobial resistance for each processed strain. These tools were also employed to detect mixed infections.

### Phylogenetic and minimum spanning tree construction

Whole-genome single nucleotide polymorphisms (wgSNPs) were extracted from the assemblies utilizing kSNP v4.0 (31). A k-mer size of 21 bp was applied, and the alignments of wgSNPs were utilized to construct a maximum likelihood (ML) phylogenetic tree using RAxML (32). The nucleotide substitution model used was a general time reversible (GTR), with 100 bootstrap estimates, and the tree was visualized using iTOLv6 (33). Additionally, GrapeTree software (34) was employed to generate and visualize a minimum spanning tree from the multi-fasta formatted SNP output obtained from MTBseq.

### Data analysis

The study results were presented using descriptive statistics. In this context, “clustered” referred to two or more isolates with identical spoligotyping patterns and <12 SNPs using spoligotyping and WGS, respectively. Conversely, “unique” denoted isolates with no common patterns and >12 SNPs. The clustering rate and RTI were calculated employing the formulas proposed by Small et al. (25). The clustering rate was determined using the formula n/N, and the RTI was calculated as (n-c)/N, where n equals the total number of clustered isolates, c is the number of clusters, and N represents the total number of isolates (25, 35).

## Results

### Clinical isolates and sequencing data quality

Out of the 96 isolates subjected to sequencing, 89 genomes met the quality criteria for genetic diversity and drug resistance analysis. Seven isolates were excluded from the analysis for the following reasons: two exhibited technical errors attributed to adapter sequences, two were identified as nontuberculous mycobacteria (NTM), and three isolates were found to consist of mixed infections. Among the 92 isolates (including mixed infections), 83 (90.22%) were collected from lymph nodes, six (6.52%) were isolated from skin lesions, one was from the abdominal area, and the remaining two (2.17%) were obtained from breast abscesses (Supplementary Table S1). For the clustering rate calculation, 87 WGS datasets were analyzed, with isolates not meeting the above quality criteria and having a mean coverage below 20× being excluded (Supplementary Table S2). Detailed characteristics of the participants and features of the MTB genome are presented in Supplementary Tables S1, S2, respectively.

### Population structure of MTB in samples from western Ethiopia

**Figure 2:**
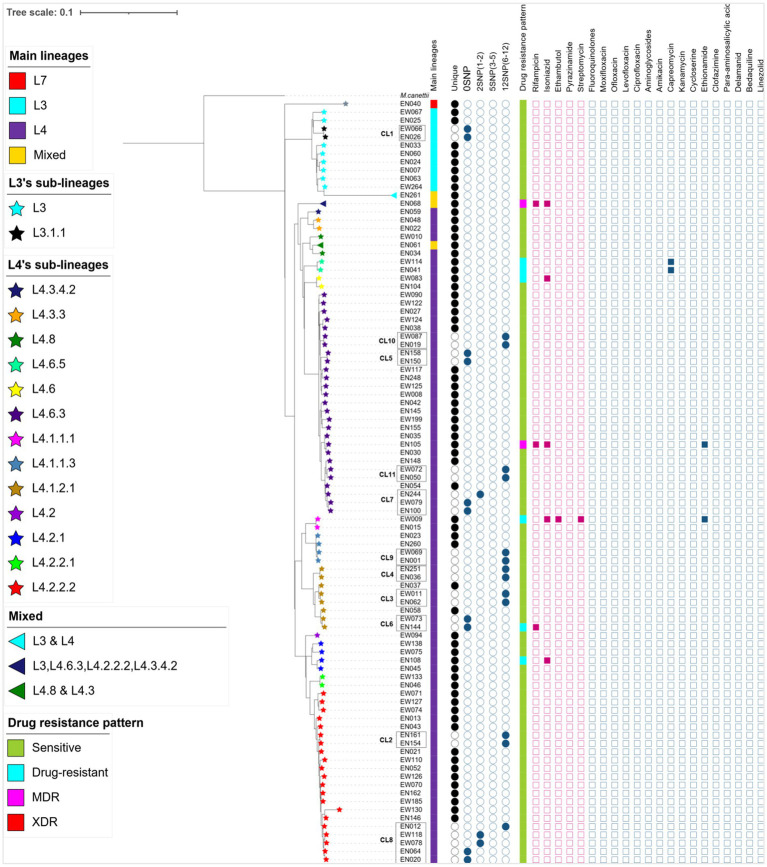
A maximum likelihood phylogenetic tree, lineage classification, clustering rate, and drug resistance patterns generated from 89 MTB isolates and 3 mixed infections collected from western Ethiopia. The lineage/sublineages, cluster assignment, and genotypic resistance to first- and second-line antituberculosis drugs were presented. The tree was annotated using iTOL v6 (33). The star symbol on the tip of each branch represents sub-lineages of MTB and the triangle shows the mixed infections. The first column denotes the main lineages. The next 5 columns show genomic relatedness within clusters (CL12, CL5, CL2, and CL0 set the maximum number of SNPs that differ from the genetically closest isolate). Next to 5 columns of the cluster, the drug resistance pattern was depicted with different colors, and mutations encoded resistance are represented by the filled square (presence of mutation) or empty square (absence of mutation) icons. The red squares are for first-line anti-TB whereas the light blue squares are for second-line drugs. To calculate cluster rate, only 87 WGS data were used; isolates consisting of mixed infections and with a mean coverage of below 20× were excluded. MDR, multi-drug resistant; XDR, extensively drug-resistant, drug-resistant (including mono and poly-resistant); L, lineage; CL, cluster.

The WGS data analysis identified 28,154 informative SNPs that differentiated among the 92 MTB strains. These SNPs were used to calculate a maximum likelihood phylogeny based on a concatenated SNP alignment (Figure 2). Based on these SNP signatures, the 92 strains were classified into three main MTBC lineages: L3, L4, and L7 (Figure 2). Notably, three isolates (EN061, EN068, and EN261) exhibit a combination of sub-lineages: EN061 demonstrates a blend of L4.8 and L4.3, EN068 manifests a mixture involving L3, L4.6.3, L4.2.2.2, and L4.3.4.2, while EN261 showcases a coexistence of L3 and L4 (Figure 2). This information underscores the complexity of genetic variation within the MTB population under study.

**Figure 3:**
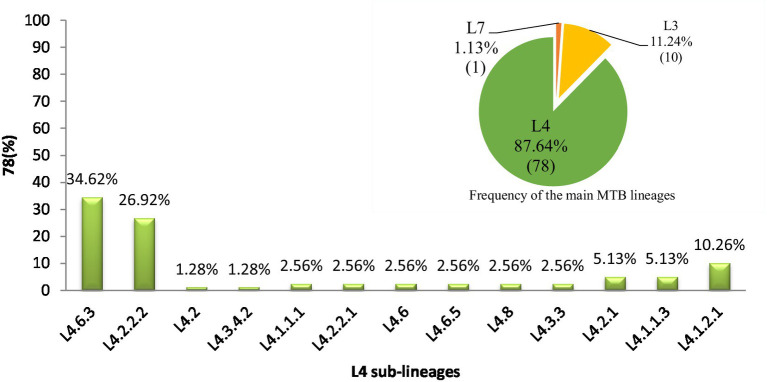
Distribution of major MTB lineages and L4 sublineages in western Ethiopia. A pie chart in the top right corner denoting the distribution of MTB main lineages in the study area and the numbers in each partition of the chart indicate the percentage of lineages calculated (L4 = green, L3 = yellow, L7 = orange). A bar graph denoting the distribution of MTB L4 sublineages and the numbers on top of the bars indicate the percentage of sub-lineages. L, lineage.

In western Ethiopia, L4 emerged as the predominant lineage, constituting 87.64% (78 out of 89 isolates), followed by L3 at 11.24%. The remaining 1.13% of the isolates belonged to L7 (Figure 3). Additionally, the isolates underwent further classification into sublineages. Within L3, there were two sub-lineages identified: L3 and L3.1.1 (Figure 2). L4 was subdivided into 13 distinct sub-lineages. Notably, L4.6.3 and L4.2.2.2 emerged as the predominant sub-lineages, constituting 34.62 and 26.92% of the total, respectively (Figures 2, 3). This detailed sub-lineage classification enhances our understanding of the genetic diversity within the main lineages and sublineages of the MTB isolates.

### *In silico* spoligotyping

The results of *in silico* spoligotyping are summarized in Table 1. According to the spoligotyping findings, 89.89% (80 out of 89) of the isolates were classified into 26 shared types (SIT numbers), while the remaining nine isolates (10.11%) were categorized as orphans. Among L3 strains, the most prevalent spoligotype was SIT 25, while among L4 strains, SIT 149 and SIT 37 were the dominant spoligotypes (Table 1).

**Table 1:** Spoligotype patterns of clustered MTB isolates (*n* = 89) of EPTB patients in western Ethiopia.

Octal code	Binary code	SIT	Main line ages	Family	*n* (%)
777777777760771	■■■■■■■■■■■■■■■■■■■■■■■■■■■■■■■■□□□□■■■■■■■	53	EA	T1	6(6.74)
777777777760751	■■■■■■■■■■■■■■■■■■■■■■■■■■■■■■■■□□□□■■■■□■■	612	EA	T1	1(1.12)
777777777760731	■■■■■■■■■■■■■■■■■■■■■■■■■■■■■■■■□□□□■■■□■■■	52	EA	T2	1(1.12)
777777777420771	■■■■■■■■■■■■■■■■■■■■■■■■■■■■□□□■□□□□■■■■■■■	777	EA	Ural-1	2(2.25)
777777777420731	■■■■■■■■■■■■■■■■■■■■■■■■■■■■□□□■□□□□■■■□■■■	817	EA	Ural-1	2(2.25)
777777775760771	■■■■■■■■■■■■■■■■■■■■■■■■■□■■■■■■□□□□■■■■■■■■	122	EA	T1	2(2.25)
777777607760771	■■■■■■■■■■■■■■■■■■■□□□□■■■■■■■■■□□□□■■■■■■■	42	EA	LAM9	1(1.12)
777777606760701	■■■■■■■■■■■■■■■■■■■■□□□□■■□■■■■■□□□□■■■□□□■	Orphan	EA	Not defined	1(1.12)
777777405760771	■■■■■■■■■■■■■■■■■■■□□□□□■□■■■■■■□□□□■■■■■■■	Orphan	EA	Not defined	2(2.25)
777777404760771	■■■■■■■■■■■■■■■■■■■□□□□□■□□■■■■■□□□□■■■■■■■	41	EA	Turkey	2(2.25)
777776777760731	■■■■■■■■■■■■■■■■■□■■■■■■■■■■■■■■□□□□■■■□■■■	336	EA	X1	4(4.49)
777776777760601	■■■■■■■■■■■■■■■■■□■■■■■■■■■■■■■■□□□□■■□□□□■	137	EA	X2	2(2.25)
777737777760771	■■■■■■■■■■■■□■■■■■■■■■■■■■■■■■■■□□□□■■■■■■■	37	EA	T3	13(14.6)
777737777760731	■■■■■■■■■■■■□■■■■■■■■■■■■■■■■■■■□□□□■■■□■■■	73	EA	T	1(1.12)
777737777760371	■■■■■■■■■■■■□■■■■■■■■■■■■■■■■■■■□□□□■■■■■■	2,040	EA	T3	1(1.12)
777737743760771	■■■■■■■■■■■■□■■■■■■■■■□□□■■■■■■■□□□□■■■■■■■	2,550	EA	Cameroon	1(1.12)
777737377760771	■■■■■■■■■■■■□■■■■■□■■■■■■■■■■■■■□□□□■■■■■■■	442	EA	T	1(1.12)
777737374020771	■■■■■■■■■■■■□■■■■■□■■■■■■□□□□□□■□□□□■■■■■■■	3,330	EA	H1	2(2.25)
777720007760771	■■■■■■■■■■■■□■□□□□□□□□□□■■■■■■■■□□□□■■■■■■■	3,341	EA	LAM-RUS	1(1.12)
777000377760771	■■■■■■■■■□□□□□□□□□□■■■■■■■■■■■■■□□□□■■■■■■■	149	EA	T3-ETH	16(17.98)
776737777760771	■■■■■■■■□■■■□■■■■■■■■■■■■■■■■■■■□□□□■■■■■■■	3,137	EA	T3	7(7.87)
776737377760771	■■■■■■■■□■■■□■■■■■□■■■■■■■■■■■■■□□□□■■■■■■■	3,324	EA	T	2(2.25)
762737777740771	■■■■■□□■□■■■□■■■■■■■■■■■■■■■■■■□□□□□■■■■■■■	Orphan	EA	Not defined	1(1.12)
703777740003471	■■■□□□□■■■■■■■■■■■■■■■□□□□□□□□□□□□■■■□□■■■■	247	EAI	CAS1-Delhi	1(1.12)
703777740003171	■■■□□□□■■■■■■■■■■■■■■■□□□□□□□□□□□□■■□□■■■■■	25	EAI	CAS1-Delhi	6(6.74)
703777700003771	■■■□□□□■■■■■■■■■■■■■■□□□□□□□□□□□□□■■■■■■■■■	142	EAI	CAS1-Delhi	1(1.12)
703377400001771	■■■□□□□■■□■■■■■■■■■□□□□□□□□□□□□□□□□■■■■■■■■	21	EAI	CAS1-Kili	2(2.25)
700000007177771	■■■□□□□□□□□□□□□□□□□□□□□□■■■□□■■■■■■■■■■■■■■	910	L7	Ethiopian	1(1.12)
600000077760731	■■□□□□□□□□□□□□□□□□□□□■■■■■■■■■■■□□□□■■■□■■■	Orphan	EA	Not defined	1(1.12)
577000377760771	■□■■■■■■■□□□□□□□□□□■■■■■■■■■■■■■□□□□■■■■■■■	Orphan	EA	T3-ETH	1(1.12)
377777607600771	□■■■■■■■■■■■■■■■■■■■□□□□■■■■■□□□□□□□■■■■■■■	Orphan	EA	Not defined	1(1.12)
376777737760771	□■■■■■■■□■■■■■■■■■■■■□■■■■■■■■■■□□□□■■■■■■■	3,315	EA	T1	1(1.12)
203436777740771	□■□□□□□■■■□□□■■■■□■■■■■■■■■■■■■□□□□□■■■■■■■	Orphan	EA	Not defined	1(1.12)
000000007760771	□□□□□□□□□□□□□□□□□□□□□□□□■■■■■■■■□□□□□■■■■■■■	Orphan	EA	Not defined	1(1.12)

EA, Euro-American; EAI, East-African-Indian; L7, lineage 7; SIT, Spoligo International Type.

Moreover, the SITVIT analysis facilitated the identification of 16 major genotypic families, with T3 representing the predominant family at 23.6% (21 out of 89), followed by the T3-ETH family, comprising 19.1% (17 out of 89) of the isolates. Intriguingly, 8.99% (8 out of 89) of the strains corresponded to spoligotypes not previously documented in the SITVIT2 database (Table 1).

### Recent disease transmission through cluster analysis

The cluster analysis revealed the presence of 11 distinct clusters, each comprised of two to five isolates, resulting in an overall clustering rate of 30% and an RTI of 17.24% (where *n* = 26, c = 11, and *N* = 87; Figure 2). The SNP differences between isolates within these clusters ranged from zero to 12. Among these clusters, only one isolate from the clustered strain (CL6) exhibited resistance to the tested TB drugs. Notably, the majority of the clustered strains belonged to L4, with a few from L3. Specifically within L4, the predominant sub-lineages were L4.6.3 and L4.2.2.2 (Figure 2).

**Figure 4:**
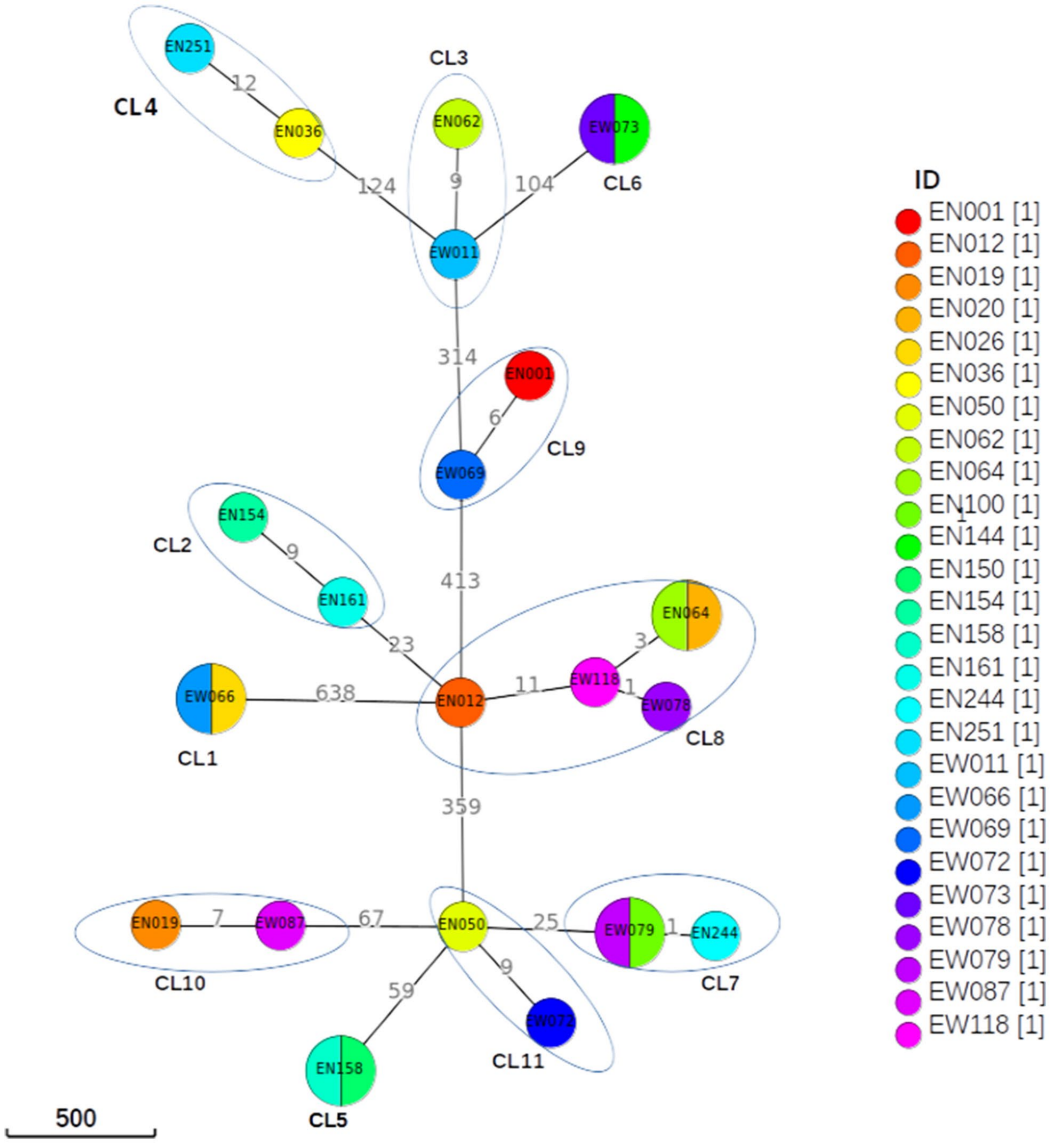
Minimum spanning tree based on SNP differences between the strains collected in western Ethiopia. The maximum distance is set to 12 SNPs for linked transmission. A distant matrix was generated from MTBseq v1.0.4 (24) and a minimum spanning tree was constructed using GrapeTree (34) software. The number indicates the SNP differences that exist between the two isolates/clusters. CL, cluster.

A difference of 0–2 SNPs between patients was observed in 13 cases (Figure 2, CL1, CL5, CL6, CL7, CL8), indicating recent transmission events. On the other hand, a difference of 6–12 SNPs was documented in 13 patients, suggesting older transmission events (Figure 2, CL5). The genetic distance between individual clusters ranged from 23 SNPs (between CL5 and CL6) to 638 SNPs (between CL3 and CL1; Figure 4). These findings provide insights into the dynamics of transmission events and genetic relationships within the MTB population under study.

### Mutations associated with drug-resistant tuberculosis

In this study, 7.87% of the isolates exhibited resistance to at least one first-line and/or second-line anti-TB drug, with only 1.12% classified as MDR isolates. Isoniazid had the highest predicted resistance, with 4.49% of the isolates demonstrating resistance to it. Additionally, 2.25% of the isolates showed resistance to rifampicin, while 1.12% exhibited resistance to each of streptomycin and ethambutol. The sequencing analysis identified a total of 11 mutations known to confer resistance to first- and second-line anti-TB drugs across seven genes (Table 2).

**Table 2:** Drug resistance pattern and associated resistance-conferring mutation of MTB in western Ethiopia (*n* = 89).

Drug	Gene	Locus tag	Genome position	Mutations	Variant type	*n* (%)
Rifampicin	*rpoB*	*Rv0667*	761135	Leu443Phe	Missense_variant	2(2.25)
			761155	Ser450Leu		
Isoniazid	*katG*	*Rv1908c*	2155700	Asn138His	Missense_variant	4(4.49)
			2155168	Ser315Thr		
			2154841	Ala424Val		
			2154973	Thr380Ile		
Isoniazid, Ethionamide	*fabG1*	*Rv1483*	1673425	−15C > T	Upstream_gene_variant	1(1.12)
Streptomycin	*rpsL*	*Rv0682*	781821	Lys88Gln	Missense_variant	1(1.12)
Ethambutol	*embB*	*Rv3795*	4248003	Gln497Arg	Missense_variant	1(1.12)
Capreomycin	*tlyA*	*Rv1694*	1918647	Asn236Lys	Missense_variant	2(2.25)
Ethionamide	*ethA*	*Rv3854c*	4326765	708delC	Frameshift_variant	2(2.25)

Remarkably, one isolate (EN068) presented with mixed infection and multidrug resistance, featuring resistance mutations in the *rpoB* (Asn437Thr, Ser441Ala, Leu464Met) and *katG* (Asn218Lys) genes (Supplementary Table S3; Figure 2). Importantly, all resistant isolates, both to first-line and second-line drugs, belonged to L4 (Figure 2). These findings underscore the genetic basis of drug resistance within the L4 lineage and highlight the importance of ongoing surveillance and management strategies to address drug-resistant TB.

## Discussion

The current study stands as a pioneering effort, utilizing WGS to explore the genetic diversity of MTB isolates, identify mixed infections, analyze drug-resistant mutations, and unveil actively ongoing transmission chains in cases of EPTB from western Ethiopia. The adoption of WGS is noteworthy as it is increasingly recognized as a valuable tool for aiding epidemiological investigations, clinical diagnosis, and control programs, contributing to decision-making in the management of infectious diseases. Despite the widespread use of WGS in developed countries, its application as a diagnostic and disease surveillance tool is slowly gaining traction in low-resource and high-tuberculosis burden settings (36, 37). This study, by employing WGS in the context of EPTB, not only fills a critical gap in the existing literature but also showcases the potential of advanced genomic techniques in enhancing our understanding of TB dynamics in resource-constrained regions.

The WGS results indicate that strains of L4 and L3 constituted 87.64 and 11.24%, respectively, of the isolates causing EPTB in western Ethiopia. This observation aligns with findings from previous studies in Ethiopia that utilized spoligotyping (38, 39), MIRU-VNTR (40), and WGS (41–43). Furthermore, the predominance of L4 as a lineage causing EPTB has been documented in neighboring countries of Ethiopia, such as Kenya (44) and Sudan (45), as well as in other African nations like Sierra Leone (46), Botswana (47), Sothern Africa (48). The prevalence of L4 MTB in Ethiopia is not unexpected, as this lineage is commonly found circulating in several countries. The success of L4 strains in Ethiopia might be attributed to their genotypic and phenotypic diversity (6, 49), their ability to evade the host immune response, and their rapid progression to TB disease, which potentially facilitates their transmission across a broader geographic scope (50, 51). Moreover, host risk factors associated with the extrapulmonary dissemination of MTB include HIV infection, younger age, female sex, and non-white race (52). This insight contributes to our understanding of the genetic diversity and regional prevalence of specific MTB lineages, shedding light on factors influencing their success in causing EPTB.

The prevalence of sub-lineages within L4 was notable in western Ethiopia, mirroring findings from prior studies in the country (39, 40, 42). Specifically, strains belonging to L4.6.3 and L4.2.2.2 were identified as the most prevalent genotypes in the region. According to the SITVIT2 database, 21 out of 27 L4.6.3 isolates and 19 out of 21 L4.2.2.2 isolates were classified as T3/Ethiopian_2 and T3-ETH/Ethiopian_3, respectively. These designations suggest that these isolates may be phylogeographically specific to Ethiopia, exhibiting a specialist profile. Such geographical restriction in genotypes could indicate a local adaptation of the strain to a particular human population, highlighting the concept of host-pathogen compatibility and then the more likely to transmit and cause disease in the same ethnicity (53). This insight enhances our understanding of the regional genetic diversity and adaptation of MTB strains in western Ethiopia.

Additionally, less common L4 sublineages, including L4.1.2.1, L4.8, and L4.3.4.2, were identified as globally distributed sublineages, often referred to as generalists (49, 54). The presence of L4.2/Ural was noted in high proportions in Asia and Africa but was largely absent from the Americas, earning it the designation of intermediate (49). The diverse geographic distributions of generalist and specialist sublineages may be attributed to intrinsic biological factors, extrinsic factors such as human migration, or a combination of both (49). It is plausible that these sublineages were introduced into Ethiopia during European contact through human migration and trade (55), contributing to the regional diversity of MTB strains.

The second most prevalent lineage identified in this study was L3, aligning with observations from previous studies conducted in different regions of Ethiopia (38–40, 42). It has been previously proposed that L3 strains have an evolutionary origin in South Asia. However, L3 strains have been frequently isolated from TB patients in East and North Africa (50, 56), leading to speculation about whether these strains have coevolved with and adapted to their East African hosts, potentially developing specific biological or phenotypic traits within this particular host population (56). The prevalence of L3 strains in Ethiopia might also be linked to historical and contemporary movements of people from South Asia, such as the Indian subcontinent, to East Africa, involving migration, tourism, and trade. These factors collectively contribute to the genetic diversity and distribution of MTB lineages in the region.

L7 was identified as the least prevalent lineage in the present study, constituting 1.13% of the isolates. Interestingly, L7 is geographically restricted to Ethiopia, making it a specialist lineage. Similar observations were noted in previous studies in southern Ethiopia (40) and eastern Ethiopia (39), where the proportion of L7 was low. In contrast, northern Ethiopia exhibited a higher proportion of L7 (57). This geographical variability in the prevalence of L7 suggests host-pathogen compatibility and relative specificity of strains to specific segments of the human population. It emphasizes that the distribution of Ethiopia-specific lineages moderately differs from one area to another within the country. This information holds significance for the country’s TB Control Program. Notably, about 8.99% of all MTB isolates in this study were not previously documented in the SITVIT2 database, emphasizing the need for further investigation and their inclusion in the genotype database.

TB disease can arise from either recent transmission of TB bacilli from active TB cases or reactivation of a previous infection. The clustering of two or more strains with similar genetic patterns indicates recent and active TB transmission within the community (58). Using the 12-SNP threshold, the overall clustering and RTI were determined to be 30 and 17.24%, respectively, suggesting high TB transmission in the area. This provides direct evidence that the high incidence rate of EPTB in western Ethiopia is not solely attributable to the reactivation of latent TB. Instead, the finding suggests the rapid progression of clinical illness from primary infection or a short latency period. Our findings are consistent with a recent WGS-based study conducted on TB Lymphadenitis (TBLN), which reported a cluster rate and RTI of 31.1 and 18%, respectively (41). However, the RTI observed in our study is higher than that reported in a study from southern Ethiopia (3.9%) (40) and northern Ethiopia (11.8%) (43). Furthermore, our RTI is lower than the overall clustering rate and RTI reported in previous studies from other regions of Ethiopia, including central Ethiopia (59), nationwide Ethiopia (60), and northern Ethiopia (42). The variations in TB transmission status could be attributed to differences in genotyping methods utilized; most studies employed spoligotyping and MIRU-VNTR typing, which have lower resolution power than WGS (60, 61). Additionally, differences in circulating MTB strains, study populations, types of TB patients (drug-susceptible vs. MDR or XDR-TB), and geographical locations could also contribute to these variations (62).

Early detection of resistance to anti-TB drugs is crucial for the successful treatment and control of drug-resistant TB (63). In this study, 7.87% of MTB strains were found to be resistant to at least one anti-TB drug, with 1.12% being MDR strains. This proportion of MDR strains is higher than those previously reported in southern Ethiopia (0.8%) (64). However, higher proportions of MDR strains have been reported in central Ethiopia (61.9%) (65), east Ethiopia (10.2%) (66), southwest Ethiopia (10.2%) (67), and at the national level in Ethiopia (11.6%) (68). The variations in resistance rates observed in our study and previous studies in Ethiopia could be attributed to differences in molecular drug resistance testing tools, virulence of circulating MTB strains in the respective geographic areas, strength of TB control programs at the study sites, various clinical characteristics of the patients (such as history of previous TB treatment, cigarette smoking, treatment compliance), economic status (nutritional status) of the patients, and immunological status of the patients (43, 69).

A total of 11 mutations, spanning across 7 genes known to confer resistance to both first- and second-line drugs used in TB treatment, were identified. Within the *katG* gene, mutations conferring resistance to isoniazid were found at various codons (Asn138His, Ala424Val, Ser315Thr, and Thr380Ile), along with the -15C > T mutation in the *fabG1* gene. All mutations except for the Ser315Thr variant were undetectable using LPA. This finding aligns with previous studies that utilized WGS to identify mutations in the *katG* gene (S315T) (43, 70–72), T380I (71), and the *fabG1* (-15C > T) (43, 70–72), which are associated with isoniazid resistance. For rifampicin resistance, mutations (Leu443Phe and Ser450Leu) in the *rpoB* gene were found, in line with other WGS studies (43). Similar to our study, additional studies have documented the presence of Lys88Gln mutation in the *rpsL* gene (71, 72), associated with streptomycin resistance, and Gln497Arg mutation in the *embB* gene (71), linked to ethambutol resistance.

In western Ethiopia, mutations linked to resistance against second-line anti-TB drugs have been identified. Specifically, ethionamide-resistance conferring mutations were observed in the *ethA* and *fabG1* genes, namely 708delC (2.25%) and -15C > T (1.12%), respectively. Notably, all isolates resistant to ethionamide in this study also exhibited resistance to isoniazid. Consistent with prior research (43, 73), the *fabG1* gene’s -15C > T mutation, responsible for resistance to both isoniazid and ethionamide, was detected in the MDR-TB isolates. Moreover, we identified missense mutations occurring at codon N236K of the *tlyA* gene in two isolates, conferring resistance to capreomycin. This mutation has also been reported in previous studies (43, 71, 74). It’s worth noting that mutations in the *ethA*, *fabG1*, and *tlyA* genes cannot be detected by LPA.

Drug-resistance tests for first- and second-line anti-TB drugs using LPA only detected resistance to rifampicin, isoniazid, fluoroquinolones, and second-line injectable drugs (amikacin, kanamycin, and capreomycin) in common resistance-conferring regions (75). However, in this study, including mutations from mixed infections, 68.75% of resistance mutations to first- and second-line anti-TB drugs were not detected by GeneXpert MTB/RIF and LPA. This observation is consistent with findings from previous studies conducted in northern Ethiopia (43, 76). Thus, this study confirmed the utility of WGS in the surveillance of drug-resistant TB strains circulating in western Ethiopia. Sequencing data analysis identified complete resistance profiles of MDR and XDR strains of MTB, suggesting that the implementation of this methodology in routine diagnostics could improve TB control at the national level. However, our study is subject to certain limitations. One such limitation is the lack of collected social-demographic information for the enrolled subjects, which prevented us from establishing epidemiological links among clustered individuals. Additionally, due to limited funding, only a small number of EPTB isolates were sequenced. Consequently, these samples may not accurately represent the region, potentially impacting the genetic diversity, drug resistance profiling, and cluster analysis.

## Conclusions and future directions

Our study reveals that MTBC strains isolated from EPTB patients in western Ethiopia display limited inter-lineage diversity, with three main lineages identified, of which L4 predominates, exhibiting high intra-lineage diversity. Among the 13 L4 sublineages identified, L4.6.3 and L4.2.2.2 are the prevailing genotypes. The elevated clustering rate and recent transmission index underscore the considerable TB transmission in the region, emphasizing the need to enhance TB infection control to disrupt the transmission chain. Although the burden of MDR-TB is low, the high transmission rate suggests potential factors such as non-genetic-based mechanisms of drug resistance (cell envelope, efflux systems, and drug degradation and modification), MTB virulence, and host risk factors. The majority of resistance-conferring mutations identified went undetected by GeneXpert MTB/RIF and LPA. Therefore, to achieve comprehensive drug-susceptibility testing, adopting WGS as a high-resolution tool for TB clinical diagnosis, detecting drug resistance mutations, and conducting molecular epidemiological analysis is crucial. This approach has the potential to significantly enhance TB control efforts and align with the WHO TB End Strategy.

### Ethical clearance

The study received ethical approval from the Addis Ababa University, Aklilu Lemma Institute of Pathobiology Institutional Review Board (ALIPB/IRB/011/2017/2018). Before sample collection, informed consent was obtained from each participant involved in the study. The study was conducted in compliance with applicable guidelines and regulations.

## Data availability statement

The datasets presented in this study can be found in online repositories. The names of the repository (e.g., NCBI) and accession number(s) can be found at: PRJNA1056148 (SRA).

## Ethics statement

The studies involving humans were approved by the Addis Ababa University, Aklilu Lemma Institute of Pathobiology Institutional Review Board (ALIPB/IRB/011/2017/2018). The studies were conducted in accordance with the local legislation and institutional requirements. Written informed consent for participation in this study was provided by the participants’ legal guardians/next of kin.

## Author contributions

BC: Conceptualization, Data curation, Formal analysis, Investigation, Methodology, Software, Validation, Visualization, Writing – original draft, Writing – review & editing. HS: Formal analysis, Software, Validation, Writing – review & editing. NG-J: Supervision, Writing – review & editing. SV: Supervision, Writing – review & editing. RW-R: Investigation, Writing – review & editing. CD: Investigation, Writing – review & editing. MG: Investigation, Writing – review & editing. OK: Supervision, Writing – review & editing. BG: Supervision, Writing – review & editing, Project administration. GA: Funding acquisition, Project administration, Supervision, Writing – review & editing.
