# Supplementary material for: Whole-genome sequencing-based genetic diversity, transmission dynamics, and drug-resistant mutations in Mycobacterium tuberculosis isolated from extrapulmonary tuberculosis patients in western Ethiopia
Source: Front Public Health. 2024 Aug 9;12:1399731. doi: 10.3389/fpubh.2024.1399731 (PMC11341482; doi:10.3389/fpubh.2024.1399731)
Supplement: Supplementary file 1 [file Table_1.DOCX]

Whole-genome sequencing-based genetic diversity, transmission dynamics, and drug-resistant mutations in *Mycobacterium* *tuberculosis* isolated from extrapulmonary tuberculosis patients in western Ethiopia

Chekesa *et al*.

Corresponding author’s emails: [balchachekesa@gmail.com](mailto:balchachekesa@gmail.com) and gobena.ameni@uaeu.ac.ae

**Table S1.** Sociodemographic characteristics of participants enrolled in the study in western Ethiopia (n=92).

| No. | ID | Age | Sex | Previous TB history | Ethnicity | Marital status | Occupation | Residence | Education level | The site of the specimen collected |
| --- | --- | --- | --- | --- | --- | --- | --- | --- | --- | --- |
| 1 | EN001 | 65 | F | No | Oromo | Married | No work | Urban | Illiterate | Lymph nodes |
| 2 | EN007 | 25 | F | No | Oromo | Married | Teacher | Urban | Collage | Lymph nodes |
| 3 | EN012 | 18 | M | No | Oromo | Unmarried | Farmer | Rular | Primary school | Lymph nodes |
| 4 | EN013 | 65 | M | No | Oromo | Married | Farmer | Rular | Illiterate | Lymph nodes |
| 5 | EN015 | 12 | M | No | Oromo | Unmarried | Student | Rular | Primary school | Skin |
| 6 | EN019 | 32 | F | No | Oromo | Married | Teacher | Urban | Degree | Lymph nodes |
| 7 | EN020 | 45 | M | No | Oromo | Married | Farmer | Rular | Illiterate | Lymph nodes |
| 8 | EN021 | 12 | M | No | Oromo | Unmarried | No work | Rular | Illiterate | Lymph nodes |
| 9 | EN022 | 30 | F | No | Oromo | Married | Farmer | Rular | Illiterate | Lymph nodes |
| 10 | EN023 | 9 | M | No | Oromo | Single | No work | Rular | Illiterate | Lymph nodes |
| 11 | EN024 | 20 | F | No | Oromo | Married | Housewife | Rular | Illiterate | Lymph nodes |
| 12 | EN025 | 35 | F | No | Oromo | Married | Farmer | Rular | Illiterate | Lymph nodes |
| 13 | EN026 | 60 | F | Yes | Oromo | Married | No work | Urban | Illiterate | Abdominal |
| 14 | EN027 | 18 | F | No | Oromo | Unmarried | Student | Rular | Primary school | Lymph nodes |
| 15 | EN030 | 21 | F | No | Oromo | Married | Housewife | Urban | Primary school | Lymph nodes |
| 16 | EN033 | 10 | F | No | Oromo | Unmarried | Student | Rular | Primary school | Lymph nodes |
| 17 | EN034 | 15 | F | No | Oromo | Unmarried | Student | Rular | Primary school | Lymph nodes |
| 18 | EN035 | 45 | F | No | Oromo | Married | Farmer | Rular | Illiterate | Lymph nodes |
| 19 | EN036 | 60 | F | NA | Oromo | Married | Farmer | Rular | Secondary school | Skin |
| 20 | EN037 | 27 | F | No | Oromo | Married | Merchant | Urban | Primary school | Lymph nodes |
| 21 | EN038 | 27 | F | No | Oromo | Married | Farmer | Rular | Primary school | Lymph nodes |
| 22 | EN040 | 65 | F | No | Oromo | Married | Farmer | Rular | Illiterate | Lymph nodes |
| 23 | EN041 | 25 | M | No | Oromo | Unmarried | Merchant | Urban | Collage | Lymph nodes |
| 24 | EN042 | 18 | F | No | Oromo | Single | Student | Rular | Secondary school | Lymph nodes |
| 25 | EN043 | 30 | M | No | Oromo | Married | Govn.t worker | Urban | Collage | Lymph nodes |
| 26 | EN045 | 48 | M | No | Oromo | Married | Farmer | Rular | Illiterate | Lymph nodes |
| 27 | EN046 | 25 | F | No | Oromo | Unmarried | No work | Rular | Collage | Lymph nodes |
| 28 | EN048 | 15 | F | No | Oromo | Unmarried | Student | Rular | Primary school | Lymph nodes |
| 29 | EN050 | 11 | F | No | Oromo | Unmarried | No work | Rular | Illiterate | Skin |
| 30 | EN052 | 15 | F | No | Oromo | Unmarried | Student | Rular | Primary school | Lymph nodes |
| 31 | EN054 | 32 | M | No | Amhara | Married | Farmer | Rular | Illiterate | Lymph nodes |
| 32 | EN058 | 21 | F | No | Other | Married | Farmer | Rular | Illiterate | Lymph nodes |
| 33 | EN059 | 22 | F | No | Oromo | Married | Merchant | Urban | Primary school | Lymph nodes |
| 34 | EN060 | 60 | M | No | Oromo | Married | Farmer | Rular | Illiterate | Lymph nodes |
| 35 | EN061 | 20 | F | No | Oromo | Married | Farmer | Rular | Illiterate | Lymph nodes |
| 36 | EN062 | 47 | F | No | Oromo | Married | Farmer | Rular | Illiterate | Lymph nodes |
| 37 | EN063 | 38 | M | No | Oromo | Married | Govn.t worker | Rular | Collage Degree | Lymph nodes |
| 38 | EN064 | 35 | M | No | Oromo | Married | Farmer | Rular | Illiterate | Lymph nodes |
| 39 | EN100 | NA | M | No | Oromo | Unmarried | Student | Rular | Secondary school | Lymph nodes |
| 40 | EN104 | 35 | F | No | Oromo | Married | Govn.t worker | Urban | Degree | Lymph nodes |
| 41 | EN105 | 17 | M | No | Amhara | Unmarried | Student | Urban | secondary school | Lymph nodes |
| 42 | EN108 |  | F | No | Oromo | Unmarried | Student | Rular | Secondary school | Lymph nodes |
| 43 | EN144 | 33 | M | No | Oromo | Married | Govn.t Worker | Rular | Secondary school | Lymph nodes |
| 44 | EN145 | 51 | F | No | Oromo | Married | Farmer | Rular | Illiterate | Skin |
| 45 | EN146 | 26 | F | No | Oromo | Married | Govn.t worker | NA | Primary school | Lymph nodes |
| 46 | EN148 | 20 | F | No | Oromo | Unmarried | Farmer | Rular | Secondary school | Lymph nodes |
| 47 | EN150 | 31 | M | No | Oromo | Married | No work | Urban | Secondary school | Lymph nodes |
| 48 | EN154 | 45 | M | No | Oromo | Married | Farmer | Rular | Illiterate | Lymph nodes |
| 49 | EN155 | 29 | M | No | Oromo | Married | Govn.t worker | Urban | Secondary school | Lymph nodes |
| 50 | EN158 | 18 | F | No | Oromo | Single | Merchant | Urban | Primary school | Lymph nodes |
| 51 | EN161 | 28 | M | No | Oromo | Married | Farmer | Rular | Primary school | Lymph nodes |
| 52 | EN162 | 52 | M | No | Amhara | Married | Farmer | Rular | Illiterate | Lymph nodes |
| 53 | EN244 | 43 | F | No | Oromo | Married | Farmer | Rular | Illiterate | Breast |
| 54 | EN248 | 18 | F | No | Oromo | Unmarried | Farmer | Rular | Secondary | Lymph nodes |
| 55 | EN251 | 17 | F | No | Oromo | Unmarried | Farmer | Rular | Primary school | Lymph nodes |
| 56 | EN260 | 27 | F | No | Oromo | Married | Farmer | Rular | Primary school | Lymph nodes |
| 57 | EN261 | 34 | M | No | Oromo | Married | Farmer | Rular | Illiterate | Lymph nodes |
| 58 | EW008 | 18 | F | No | Oromo | Unmarried | Student | Rular | High school | Lymph nodes |
| 59 | EW009 | 20 | M | No | Oromo | Married | No work | Urban | Illiterate | Lymph nodes |
| 60 | EW010 | 21 | F | No | Oromo | Married | Farmer | Urban | Illiterate | Lymph nodes |
| 61 | EW011 | 26 | F | No | Amhara | Unmarried | Student | Urban | High school | Lymph nodes |
| 62 | EW066 | 31 | M | No | Oromo | Married | Farmer | Urban | Primary school | Lymph nodes |
| 63 | EW067 | 24 | M | No | Oromo | Married | Farmer | Rular | Primary school | Lymph nodes |
| 64 | EW068 | 22 | M | No | Oromo | Married | Student | Rular | Collage | Skin |
| 65 | EW069 | 25 | M | No | Amhara | Married | Farmer | Rular | Primary school | Lymph nodes |
| 66 | EW070 | 2 | M | No | Oromo | Unmarried | No work | Urban | Illiterate | Lymph nodes |
| 67 | EW071 | 30 | F | No | Oromo | Married | Teacher | Urban | Degree | Lymph nodes |
| 68 | EW072 | 26 | M | Yes | Oromo | Married | Farmer | Rular | Primary school | Lymph nodes |
| 69 | EW073 | 35 | M | No | Oromo | Married | Farmer | Rular | Primary school | Lymph nodes |
| 70 | EW074 | 50 | M | No | Oromo | Married | Farmer | Rular | Illiterate | Lymph nodes |
| 71 | EW075 | 24 | F | No | Oromo | Unmarried | Cleaner | Urban | Collage | Lymph nodes |
| 72 | EW078 | 48 | F | Yes | Oromo | Married | Farmer | Rular | Illiterate | Lymph nodes |
| 73 | EW079 | 45 | M | Yes | Oromo | Married | Farmer | Rular | Illiterate | Lymph nodes |
| 74 | EW083 | 65 | M | No | Oromo | Married | Farmer | Rular | Illiterate | Lymph nodes |
| 75 | EW087 | 15 | F | No | Oromo | Unmarried | Farmer | Rular | Illiterate | Lymph nodes |
| 76 | EW090 | 19 | F | No | Oromo | Single | Student | Urban | Secondary school | Lymph nodes |
| 77 | EW094 | 26 | F | No | Amhara | Unmarried | Student | Urban | Illiterate | Breast |
| 78 | EW110 | NA | NA | No | Oromo | NA | NA | Rular | NA | Lymph nodes |
| 79 | EW114 | 18 | F | No | Oromo | Unmarried | Student | Rular | Secondary school | Lymph nodes |
| 80 | EW117 | NA | NA | No | Oromo | Married | Student | NA | NA | Lymph nodes |
| 81 | EW118 | 35 | F | No | Oromo | Married | Farmer | Rular | Illiterate | Lymph nodes |
| 82 | EW122 | 23 | M | No | Oromo | Married | Farmer | Rular | Primary school | Lymph nodes |
| 83 | EW124 | 46 | M | No | Oromo | Married | Merchant | Urban | Elementary school | Lymph nodes |
| 84 | EW125 | 27 | M | No | Oromo | Married | Farmer | Rular | Elementary school | Skin |
| 85 | EW126 | NA | NA | No | Oromo | NA | NA | NA | NA | Lymph nodes |
| 86 | EW127 | 18 | M | No | Oromo | Single | Farmer | Rular | Illiterate | Lymph nodes |
| 87 | EW130 | NA | NA | No | Oromo | NA | Student | NA | Primary school | Lymph nodes |
| 88 | EW133 | 50 | M | Yes | Oromo | Married | Farmer | Rular | Illiterate | Lymph nodes |
| 89 | EW138 | 13 | M | No | Oromo | Single | No work | Rular | Primary school | Lymph nodes |
| 90 | EW185 | 25 | M | No | Oromo | Married | Farmer | Rular | Secondary | Lymph nodes |
| 91 | EW199 | 30 | F | No | Oromo | Married | Farmer | Rular | Illiterate | Lymph nodes |
| 92 | EW264 | 19 | F | No | Oromo | Unmarried | Student | Urban | Collage | Lymph nodes |

*NA* not available

**Table S2.** Genomic features of MTB in EPTB patients in western Ethiopia (n=89).

| No. | | ID | Total Reads | Mapped Reads | % Mapped Reads | Total Bases | % (Any) Total Bases | GC-Content | Coverage mean |
| --- | --- | --- | --- | --- | --- | --- | --- | --- | --- |
| 1 | EN001 | | 8116305 | 8073748 | 99.48 | 4398015 | 1 | 65.6 | 237.61 |
| 2 | EN007 | | 6386108 | 6252369 | 97.91 | 4395904 | 1 | 65.59 | 184.08 |
| 3 | EN012 | | 4349942 | 4193825 | 96.41 | 4390415 | 1 | 65.6 | 121.29 |
| 4 | EN013 | | 7385239 | 7288500 | 98.69 | 4391993 | 1 | 65.61 | 213.73 |
| 5 | EN015 | | 35649021 | 35256579 | 98.9 | 4401365 | 1 | 65.62 | 1032.33 |
| 6 | EN019 | | 25828349 | 25749106 | 99.69 | 4405040 | 1 | 65.61 | 763.76 |
| 7 | EN020 | | 6802589 | 4759741 | 69.97 | 4388414 | 0.99 | 65.6 | 142.72 |
| 8 | EN021 | | 8506475 | 8457371 | 99.42 | 4391965 | 1 | 65.6 | 252.83 |
| 9 | EN022 | | 7038627 | 7020667 | 99.74 | 4367695 | 0.99 | 65.6 | 209.77 |
| 10 | EN023 | | 6724012 | 6690439 | 99.5 | 4397282 | 1 | 65.6 | 197.22 |
| 11 | EN024 | | 6713382 | 6678049 | 99.47 | 4392300 | 1 | 65.59 | 196.47 |
| 12 | EN025 | | 5882868 | 5166094 | 87.82 | 4400675 | 1 | 65.6 | 150.57 |
| 13 | EN026 | | 3497250 | 2583368 | 73.87 | 4396435 | 1 | 65.59 | 77.31 |
| 14 | EN027 | | 8004594 | 7357696 | 91.92 | 4401172 | 1 | 65.6 | 212.68 |
| 15 | EN030 | | 5606616 | 5507190 | 98.23 | 4402357 | 1 | 65.6 | 159.6 |
| 16 | EN033 | | 6686783 | 6084944 | 91 | 4403472 | 1 | 65.6 | 176.78 |
| 17 | EN034 | | 18676028 | 18240908 | 97.67 | 4400642 | 1 | 65.6 | 540.75 |
| 18 | EN035 | | 7538891 | 7470071 | 99.09 | 4401063 | 1 | 65.61 | 218.22 |
| 19 | EN036 | | 10172739 | 10084890 | 99.14 | 4396314 | 1 | 65.62 | 291.5 |
| 20 | EN037 | | 8389876 | 8283699 | 98.73 | 4393361 | 1 | 65.61 | 247.01 |
| 21 | EN038 | | 8281340 | 8203076 | 99.05 | 4403080 | 1 | 65.61 | 232.6 |
| 22 | EN040 | | 8304778 | 8221875 | 99 | 4375322 | 0.99 | 65.59 | 243.79 |
| 23 | EN041 | | 7047354 | 7020544 | 99.62 | 4408366 | 1 | 65.61 | 201.62 |
| 24 | EN042 | | 9231478 | 9192747 | 99.58 | 4404124 | 1 | 65.61 | 263.59 |
| 25 | EN043 | | 6603590 | 6559315 | 99.33 | 4387973 | 0.99 | 65.6 | 193.17 |
| 26 | EN045 | | 14169589 | 11877281 | 83.82 | 4398971 | 1 | 65.61 | 339.97 |
| 27 | EN046 | | 6532229 | 4081987 | 62.49 | 4396037 | 1 | 65.59 | 116.58 |
| 28 | EN048 | | 4152284 | 4074738 | 98.13 | 4389736 | 1 | 65.59 | 117.67 |
| 29 | EN050 | | 6081784 | 5654917 | 92.98 | 4403036 | 1 | 65.6 | 162.86 |
| 30 | EN052 | | 6789102 | 6740396 | 99.28 | 4390154 | 1 | 65.6 | 199.75 |
| 31 | EN054 | | 8744575 | 8712498 | 99.63 | 4400949 | 1 | 65.61 | 253.59 |
| 32 | EN058 | | 9443990 | 8689408 | 92.01 | 4390284 | 1 | 65.62 | 254.79 |
| 33 | EN059 | | 9220887 | 9200583 | 99.78 | 4357257 | 0.99 | 65.61 | 270.42 |
| 34 | EN060 | | 8230228 | 8189525 | 99.51 | 4396543 | 1 | 65.6 | 238.07 |
| 35 | EN062 | | 8299711 | 8257760 | 99.49 | 4392075 | 1 | 65.61 | 243.64 |
| 36 | EN063 | | 9083716 | 9030017 | 99.41 | 4394530 | 1 | 65.6 | 266.56 |
| 37 | EN064 | | 8340493 | 8295693 | 99.46 | 4391527 | 1 | 65.61 | 242.99 |
| 38 | EN100 | | 2258206 | 2252185 | 99.73 | 4397033 | 1 | 65.59 | 63.38 |
| 39 | EN104 | | 687821 | 684618 | 99.53 | 4391693 | 1 | 65.57 | 19.08 |
| 40 | EN105 | | 1322877 | 1315630 | 99.45 | 4399584 | 1 | 65.59 | 36.32 |
| 41 | EN108 | | 1607458 | 1594636 | 99.2 | 4390279 | 1 | 65.57 | 44.1 |
| 42 | EN144 | | 1162067 | 981959 | 84.5 | 4389104 | 0.99 | 65.58 | 26.79 |
| 43 | EN145 | | 1285045 | 1281258 | 99.71 | 4393011 | 1 | 65.59 | 35.54 |
| 44 | EN146 | | 1566468 | 1555261 | 99.28 | 4383835 | 0.99 | 65.58 | 42.77 |
| 45 | EN148 | | 9438563 | 6473427 | 68.58 | 4405531 | 1 | 65.61 | 180.72 |
| 46 | EN150 | | 20106500 | 19980350 | 99.37 | 4404392 | 1 | 65.62 | 563.42 |
| 47 | EN154 | | 9100210 | 9049303 | 99.44 | 4393151 | 1 | 65.61 | 250.19 |
| 48 | EN155 | | 1013758 | 1009311 | 99.56 | 4383657 | 0.99 | 65.57 | 28.43 |
| 49 | EN158 | | 2329835 | 2320573 | 99.6 | 4396839 | 1 | 65.59 | 66.15 |
| 50 | EN161 | | 4233128 | 4205834 | 99.36 | 4390648 | 1 | 65.61 | 116.49 |
| 51 | EN162 | | 2903662 | 2876968 | 99.08 | 4388039 | 0.99 | 65.6 | 78.94 |
| 52 | EN244 | | 7046065 | 6865125 | 97.43 | 4405743 | 1 | 65.61 | 191.89 |
| 53 | EN248 | | 3254972 | 3200080 | 98.31 | 4400025 | 1 | 65.61 | 88.99 |
| 54 | EN251 | | 1309692 | 1302775 | 99.47 | 4389422 | 0.99 | 65.6 | 35.86 |
| 55 | EN260 | | 1530074 | 1521798 | 99.46 | 4394234 | 1 | 65.59 | 42.72 |
| 56 | EW008 | | 6282292 | 4877618 | 77.64 | 4398394 | 1 | 65.6 | 142.56 |
| 57 | EW009 | | 7871616 | 7775555 | 98.78 | 4399417 | 1 | 65.61 | 225.64 |
| 58 | EW010 | | 5937751 | 4651174 | 78.33 | 4391710 | 1 | 65.59 | 135.83 |
| 59 | EW011 | | 6436282 | 6384458 | 99.19 | 4394208 | 1 | 65.61 | 185.45 |
| 60 | EW066 | | 7861806 | 7825420 | 99.54 | 4402542 | 1 | 65.6 | 227.72 |
| 61 | EW067 | | 8641834 | 5756690 | 66.61 | 4401609 | 1 | 65.6 | 169.68 |
| 62 | EW069 | | 2784627 | 2771574 | 99.53 | 4396096 | 1 | 65.6 | 77.37 |
| 63 | EW070 | | 3120925 | 3092911 | 99.1 | 4386423 | 0.99 | 65.59 | 88.76 |
| 64 | EW071 | | 781787 | 777088 | 99.4 | 4379774 | 0.99 | 65.56 | 21.77 |
| 65 | EW072 | | 1143230 | 1140270 | 99.74 | 4393711 | 1 | 65.58 | 32.63 |
| 66 | EW073 | | 1477413 | 1470879 | 99.56 | 4386707 | 0.99 | 65.6 | 40.92 |
| 67 | EW074 | | 900016 | 894799 | 99.42 | 4385769 | 0.99 | 65.56 | 25.62 |
| 68 | EW075 | | 1995478 | 1983528 | 99.4 | 4391816 | 1 | 65.58 | 56.51 |
| 69 | EW078 | | 1821411 | 1810795 | 99.42 | 4385499 | 0.99 | 65.59 | 50.91 |
| 70 | EW079 | | 862587 | 860376 | 99.74 | 4395951 | 1 | 65.58 | 23.93 |
| 71 | EW083 | | 3727657 | 3709714 | 99.52 | 4403330 | 1 | 65.6 | 103.42 |
| 72 | EW087 | | 4424555 | 4412696 | 99.73 | 4398777 | 1 | 65.6 | 124.47 |
| 73 | EW090 | | 1500162 | 1490163 | 99.33 | 4395009 | 1 | 65.59 | 41.75 |
| 74 | EW094 | | 4084790 | 4061389 | 99.43 | 4398011 | 1 | 65.6 | 116.67 |
| 75 | EW110 | | 833098 | 826861 | 99.25 | 4375381 | 0.99 | 65.58 | 23.36 |
| 76 | EW114 | | 1051504 | 1046489 | 99.52 | 4382909 | 0.99 | 65.58 | 28.81 |
| 77 | EW117 | | 1721966 | 1714922 | 99.59 | 4386702 | 0.99 | 65.56 | 48.24 |
| 78 | EW118 | | 1939649 | 1925999 | 99.3 | 4388022 | 0.99 | 65.6 | 53.95 |
| 79 | EW122 | | 1754680 | 1601172 | 91.25 | 4400200 | 1 | 65.6 | 43.83 |
| 80 | EW124 | | 849313 | 845114 | 99.51 | 4393687 | 1 | 65.58 | 23.73 |
| 81 | EW125 | | 2203826 | 2195938 | 99.64 | 4401167 | 1 | 65.6 | 61.89 |
| 82 | EW126 | | 1527611 | 1518997 | 99.44 | 4386455 | 0.99 | 65.59 | 41.13 |
| 83 | EW127 | | 1416113 | 1399361 | 98.82 | 4396618 | 1 | 65.59 | 38.78 |
| 84 | EW130 | | 680206 | 676003 | 99.38 | 4346938 | 0.99 | 65.48 | 19.67 |
| 85 | EW133 | | 1989244 | 1961340 | 98.6 | 4393598 | 1 | 65.58 | 56.67 |
| 86 | EW138 | | 1832848 | 1819597 | 99.28 | 4403677 | 1 | 65.59 | 49.58 |
| 87 | EW185 | | 3361815 | 3343365 | 99.45 | 4389275 | 0.99 | 65.6 | 91.54 |
| 88 | EW199 | | 2207063 | 2134480 | 96.71 | 4402657 | 1 | 65.6 | 60.07 |
| 89 | EW264 | | 1029898 | 1023684 | 99.4 | 4392606 | 1 | 65.58 | 27.82 |
| Average |  | | **5671059.28** | **5409585.66** | **96.1** | **4393446.16** | **0.99753** | **65.5951** | **156.633** |

| ID | Drug | Genome Position | Locus Tag | Gene | Variant Type | Change | Remark |
| --- | --- | --- | --- | --- | --- | --- | --- |
| EW114 | Capreomycin | 1918647 | Rv1694 | *tlyA* | missense_variant | p.Asn236Lys | Not mixed |
| EN144 | Rifampicin | 761135 | Rv0667 | *rpoB* | missense_variant | p.Leu443Phe | Not mixed |
| EN108 | Isoniazid | 2155700 | Rv1908c | *katG* | missense_variant | p.Asn138His | Not mixed |
| EN068 | Rifampicin | 761116 | Rv0667 | *rpoB* | missense_variant | p.Asn437Thr | Mixed |
| EN068 | Rifampicin | 761127 | Rv0667 | *rpoB* | missense_variant | p.Ser441Ala | Mixed |
| EN068 | Rifampicin | 761196 | Rv0667 | *rpoB* | missense_variant | p.Leu464Met | Mixed |
| EN068 | Isoniazid | 2155458 | Rv1908c | *katG* | missense_variant | p.Asn218Lys | Mixed |
| EN105 | Rifampicin | 761155 | Rv0667 | *rpoB* | missense_variant | p.Ser450Leu | Not mixed |
| EN105 | isoniazid,ethionamide | 1673425 | Rv1483 | *fabG1* | upstream_gene_variant | c.-15C>T | Not mixed |
| EN105 | Isoniazid | 2154973 | Rv1908c | *katG* | missense_variant | p.Thr380Ile | Not mixed |
| EN041 | Capreomycin | 1918647 | Rv1694 | *tlyA* | missense_variant | p.Asn236Lys | Not mixed |
| EW009 | Streptomycin | 781821 | Rv0682 | *rpsL* | missense_variant | p.Lys88Gln | Not mixed |
| EW009 | Isoniazid | 2155168 | Rv1908c | *katG* | missense_variant | p.Ser315Thr | Not mixed |
| EW009 | Ethambutol | 4248003 | Rv3795 | *embB* | missense_variant | p.Gln497Arg | Not mixed |
| EW009 | ethionamide,ethionamide | 4326765 | Rv3854c | *ethA* | frameshift_variant | c.708delC | Not mixed |
| EN083 | Isoniazid | 2154841 | Rv1908c | *katG* | missense_variant | p.Ala424Val | Not mixed |

**Table S3**. Resistance conferring mutations with their corresponding isolates and drugs.
